# Gut microbiota of endangered crested ibis: Establishment, diversity, and association with reproductive output

**DOI:** 10.1371/journal.pone.0250075

**Published:** 2021-04-23

**Authors:** Jian Ran, Qiu-Hong Wan, Sheng-Guo Fang

**Affiliations:** MOE Key Laboratory of Biosystems Homeostasis & Protection, State Conservation Centre for Gene Resources of Endangered Wildlife, College of Life Sciences, Zhejiang University, Hangzhou, P. R. China; University of Maine, UNITED STATES

## Abstract

Gut microbiota is known to influence the host’s health; an imbalance of the gut microbial community leads to various intestinal and non-intestinal diseases. Research on gut microbes of endangered birds is vital for their conservation. However, a thorough understanding of the gut microbiome composition present in crested ibises at different ages and its correlation with crested ibis reproductive capacity has remained elusive. Here, we used 16S rRNA gene sequencing to explore the fecal microbial structure of nestlings and adult birds, and the difference in gut microbiota between healthy and sterile crested ibises. We observed that (1) bacterial microbiota, alpha and beta diversity of one-day-old nestlings significantly distinguished from other nestlings; abundance of Proteobacteria decreased, while that of Fusobacteria increased with an increase in the age of the nestlings; (2) there was no significant difference in community composition among adult crested ibises aged one, two, three, and five years; (3) the abundance of Proteobacteria and alpha diversity indices were higher in sterile crested ibises than in healthy crested ibises; thus, Proteobacteria can act as a diagnostic biomarker of reproductive dysfunction in crested ibises. This study significantly contributes to the field of ecology and conservation, as it provides a platform for assessing the reproductive capacity of endangered crested ibises, based on the gut microbiota composition. Further studies may unravel additional factors influencing crested ibises’ reproductive health, which will further help the management and control of the crested ibis population.

## Introduction

Gut microbiota plays an integral part in the processes that maintain the host’s health, such as host metabolism, bio-antagonism, and immunity [1]. Specific microbial species reside in different parts of the gut; their abundance depends on food intake, metabolism, and genetic inheritance of the host cells [2]. Dysbiosis of intestinal flora refers to the destruction of the balance between normal microbial community and its host under the influence of different factors. Dysbiosis of intestinal flora is related to many infectious and non-infectious diseases, which can be both intestinal and extraintestinal [3]. Scientists have proposed that intestinal flora is a "neglected and forgotten organ" [4], which could be the "master" of host development and physiology [5]. Given the essential role of gut microbiota in maintaining host health, extensive research focused on intestinal microorganisms [6]; however, these studies have mainly focused on the composition and diversity of mammals and avian gut microbiota. Only a few studies have been done on the establishment process of the flora of endangered birds and the correlation between gut microbiota and reproductive output, which are important to the health and growth of the population, and which encourage the need to study the gut microbiota in birds.

The establishment and succession of intestinal flora is a complex process [7]. Various parameters, such as mode of delivery, environmental factors, dietary structure, and intake of antibiotics affect the composition of intestinal flora during the early stages of the establishment [8, 9]. Previously, a study on the intestinal flora of humans has reported that bacterial colonization of newborns begins before birth and undergoes primary succession. At the age of two or three, the intestinal flora of a child maintains a stable and dynamic balance and is similar to the microflora of an adult [10]. Inheritance and establishment of gut microbiota have also been explored in non-human organisms. A previous study revealed that intestinal flora richness of giant panda cubs increases progressively with age, especially from the age of six months [11]. In birds, there are two perspectives on the construction and succession of gut microbiota. Some researchers have demonstrated that birds acquire gut microbiota from the nesting environment after hatching and then from parental feeding [12, 13]. In contrast, scientists have also demonstrated that the microbes found in chicken embryos are similar to those in the mother-hen. This supports the hypothesis that bacteria colonize the intestinal tract of the host during embryonic development and most of the pioneer microbial species are inherited from the mother [14, 15]. After recruitment, the gut microbiota of birds gradually undergoes a series of bacterial successions. Grond et al. (2017) reported that the intestinal microbes in the arctic-breeding shorebird chicks proliferate within one to two days of hatching and become stable: Clostridia and Gammaproteobacteria being the dominant bacterial classes [12]. A study reported that a continuous dynamic change occurred in the fecal microflora of broilers from 1 to 35 days, and the composition of the community changed significantly at 3–4 days and 21 days old [16]. Although variations in microbial community structure of gut microbiota based on different organisms have been reported [17–19], there is a dearth of studies that have evaluated the succession of gut microbiota and the factors affecting it.

Ley et al. (2008) investigated the fecal microbial communities of 60 species of mammals. The dominant phyla in these mammals are Firmicutes (65.7%), Bacteroidetes (16.3%), Proteobacteria (8.8%), and Actinobacteria (4.7%) [20]. Similar to mammalian hosts, the gut microbiota of avian hosts is also dominated by these four phyla, although their relative abundance varies considerably [21]. For example, the gut microbiota of chickens has a higher abundance of Proteobacteria than that of most mammals [22]. A previous study has indicated that the gut microbiota of avian hosts clusters differently from that of mammals and insects [23]. According to community richness analyses, only 17 bacterial phyla have been observed in mammals, whereas 4 to 40 bacterial phyla have been found among different avian hosts [20, 24, 25]. In addition, gut microbiota has a close relationship with physiology and behavior of birds [18]. One study found that gut microbiota of birds weakly correlated to host phylogeny or diet. The researchers speculated that this may be due to the physiological structure of birds which adapt to flight function. Compared with mammals, birds have a shorter intestinal tract and shorter intestinal content retention times. The abundance of obligate anaerobes is less, whereas the abundance of facultative anaerobes is more in birds [26]. Furthermore, the gut microbiota will vary with a change in physiological state. The cloacal bacterial community reportedly differed between different genders under reproductive conditions. In males, the bacterial community becomes more diverse with the beginning of reproduction, and then decreases in diversity with the transition to non-breeding states [27]. In hooded crane, the alpha diversity of intestinal bacteria is significantly higher in winter than in autumn and spring, and the composition of bacterial flora in winter is significantly different from compared to autumn and spring [28]. In poultry, a significant correlation has been found between intestinal microflora and seasons [29]. In addition, the migration habits of birds also affect the intestinal microflora of birds. The intestinal microflora of songbirds become similar within species and among different species during stopovers, indicating that local diet or environment plays an important role as a potential driver of change in intestinal microbial community [18]. However, more research is required to identify the composition of gut microbiota in different avian species, and adequately demonstrate the influence of gut microbiota on host health and disease.

Equilibrium of intestinal microflora is closely related to the health of the host [30]. Previous studies have shown that gut microbiota plays an essential role in defense against infection, immunomodulation, metabolic regulation, and nutrient digestion and absorption [30–32]. Several studies have also reported the role of gut microbiota in various extraintestinal diseases, such as inflammatory bowel diseases, cardiometabolic disorders, neuropsychiatric diseases, and cancer [2]. The gut microbiome can also regulate steroid production and hormones, and affect the reproductive capacity of humans [33, 34]. For non-human hosts, scientists have revealed that the host microbiome is associated with hormone production; however, it is unclear whether their relationship is significant for reproductive output [35–38]. Antwis et al. (2019) suggested that the changes in gut microbiota could be a potential biomarker of reproductive health in the endangered eastern black rhino [39]. The dysbiosis of intestinal flora in birds can lead to intestinal dysfunction [40], but the effect of dysbiosis on non-intestinal diseases is still unknown. At the community level, the response intensity of phytohaemaglutinin (PHA) was significantly correlated with the composition of intestinal microflora in nestlings of a passerine bird, indicating the composition of bacteria was closely related to immunity [41]. Another study showed a significant correlation between microbial diversity and phenotypic traits of great spotted cuckoo *Clamator glandarius* nestlings, which suggested that bacterial community may be considered as a potential biomarker for the future survival and breeding of young birds [42]. A study in barn swallows has confirmed that the diversity of cloacal microbiomes is closely related to important individual characteristics such as survival, indicating that cloacal microbiomes should be contained among the traits assessed in bird ecological studies [43].

Crested ibis is an endangered wild bird, which was once considered extinct. Although the population of crested ibis is gradually recovering and has reached 2,600 individuals after artificial propagation [44], it is still considered an endangered species. Problems, such as severe inbreeding, low reproductive capacity, high disability rate of chicks, and frequent digestive diseases have been affecting the progress of the population [45–47].

We speculated that the composition of intestinal flora could be an important factor that influences the reproductive success and population levels in crested ibises. Thus, in this study, we investigated the gut microbiota of crested ibises to monitor its influence on reproductive system diseases and ontogenesis. We used 16S rRNA sequencing technology to identify the fecal bacterial composition of crested ibis nestlings and adults. The following three aspects were explored: (1) the establishment and variation of gut microbiota in nestlings; (2) the composition and diversity of gut microbiota in adults; (3) comparison of gut microbiota between healthy and sterile crested ibises.

## Materials and methods

### Ethics statement

All sample collection was conducted according to the national and international guidelines for animal welfare. We collected fecal samples with the permission of the Crested Ibis Breeding Centers (CIBC), Deqing County, Zhejiang Province, China. During the sampling period, non-invasive sampling was carried out to minimize fright to the crested ibises.

### Animal and sample collection

Crested ibis (Nipponia nippon) belong to Aves (Pelecaniformes, Threskiornithidae). Crested ibis is listed as endangered birds in the red list of the International Union of conservation organizations (IUCN). The breeding period of crested ibis is from February to June. A pair of crested ibis usually lays a litter per year, with 1–4 eggs. After the crested ibis laid eggs, the eggs were moved to an artificial incubator. The incubation period of crested ibis is 28 days and the umbilical cord was sterilized with iodophor after hatching. Then the newly hatched chicks were placed in incubator with temperature of 37.3°C and humidity of 50%. At 8 hours after hatching, we fed a small amount of warm water to the nestlings. At around the 10 days of age, the chicks were transferred to the brooding room. The temperature in the brooding room was 27°C– 30°C, and the humidity was 50%– 60%. At 23 days of age, the nestlings were artificially kept at the room temperature to 45 days of age, and then placed in an outdoor artificial cage. The feed composition of nestlings and adult birds was adjusted according to age **(S1 Table)**. During the breeding period from February to June, non-staff members were not allowed to contact or visit, so as to provide a good breeding environment for crested ibis.

To investigate the establishment of gut microbiota in nestlings, we randomly selected 12 healthy nestlings (six female birds and six male birds) that were artificially hatched. The fecal samples of nestlings were collected in the CIBC, Deqing County, Zhejiang Province, China, and were collected from March 11 to May 30, 2018. We collected the feces of 12 nestlings when they were 1-day-old (D01), 9-days-old (D09), 18-days-old (D18), 27-days-old (D27), 36-days-old (D36), and 45-days-old (D45). We obtained a total of 72 fecal samples from the nestlings **(S2 Table)**. The sampling was done in line with aseptic technique and the special requirements below. Fresh feces were collected immediately and stored in sterile cryotubes. We only kept the internal part of the feces and discarded the external part. After collection, feces were stored in an ice box immediately transported back to the laboratory for storage at—80°C within one hour.

To analyze the structure of gut microbiota in adult crested ibises, we collected cloacal swabs of 12 adult birds at the CIBC, Deqing County, Zhejiang Province, China, in August 15, 2018. We randomly selected three birds from each age group with a total of seven males and five females. We had four age groups 1-year (Y1), 2- years (Y2), 3-years (Y3), and 5-years (Y5) **(S2 Table)**. We inserted a sterile cotton swab into the cloaca of the crested ibis, wiped it gently and slowly rotated to stain it with excrement, and then took it out. The cotton part of the swab was placed in a 2-ml cryotube containing a sample preservation solution, PBS buffer. Samples were placed in an ice box, transported back to the laboratory and stored at -80°C until use.

To evaluate the relationship between intestinal flora and reproductive capacity, we collected cloacal swabs from sterile crested ibis (n = 3) and normal crested ibis (n = 8) on September 23, 2018. The collection method of cloacal swabs was the same as those of the 12 adults **(S2 Table)**. All the samples were stored in cryotubes at −80°C until DNA was extracted.

### DNA extraction and sequencing

Total genomic DNA was isolated from the fecal samples using the QIAamp DNA Stool Mini Kit (Qiagen) according to the manufacturer’s instructions. The V4 region of the bacterial 16S rRNA gene was amplified by the primers, 515F (GTGYCAGCMGCCGCGGTAA), and 806R (GGACTACNVGGGTWTCTAAT) and a 6-bp barcode was uniquely assigned to each sample. To ensure accuracy and check for contamination, a positive control (with ZymoBIOMICS® Microbial Community Standard) and a negative control without DNA template and equal amount of water were performed. All PCR reactions were carried out in a 30 μL mixture with 10 ng template, 0.2 μM of each primer, and 15 μL of PCR Master Mix (New England Biolabs). The PCR reaction conditions were as follows: 98°C for 1 min, followed by 30 cycles of denaturation at 98°C for 10 s, 50°C for 30 s, and 72°C for 30 s, with a final extension at 72°C for 5 min. We mixed the PCR products in equidensity ratios and purified them using GeneJET Gel Extraction Kit (Thermo Scientific). The library construction was done by NEB Next^®^ Ultra™ DNA Library Prep Kit for Illumina^®^ (New England Biolabs) following the manufacturer’s instructions. The sequencing was performed on Illumina HiSeq 2500 platform with the standard Illumina protocols. Library construction and sequencing were carried out at the Tianjin Personal Biotechnology Limited Company.

#### Sequencing data processing

*Paired-end reads assembly and quality control*. Firstly, Paired-end reads were assigned to samples based on their specific barcode and truncated by cutting off the barcode and primer sequence. Then, the paired-end reads were merged by FLASH to obtain splicing sequences which called raw data [48]. To remove low-quality reads, raw data were quality-filtered and de-multiplexed by Quantitative Insights into Microbial Ecology (QIIME2). The data filtering criteria were as follows: remove the sequence with 5’ primer mismatch base > 1; remove the sequence containing N (fuzzy base); remove the sequence with consecutive identical bases > 8; remove the sequence with length ≤ 150 bp; remove the chimeric sequence. At least, the effective sequences were finally obtained.

*OTU cluster and species annotation*. The sequences were dereplicated and clustered into operational taxonomic units (OTUs) at an identity threshold of 97% by using the UCLUST in QIIME, and select the longest sequence in each class as the representative sequence. Then, RDP classifier was used to annotate taxonomic data for the representative sequences. The annotation database is: Greengene (Release 13.8, http://greengenes.secondgenome.com/). Subsequently, OTUs with abundance less than 0.001% of the total number of sequences were removed, and OTUs abundance information was normalized using a standard of sequence number (cutoff = 36602) corresponding to the sample with the least sequences.

### Statistical analysis

The taxonomic assignment, and alpha and beta diversity analyses were performed using the quantitative insights into microbial ecology (QIIME2) software packages [49]. The core genus of crested ibises under study were defined as average relative abundance > 0.1% [22]. To compare alpha diversity, five metrics were calculated: Chao1 and Ace indexes estimated the community abundance; observed species showed the amount of unique OTUs found in each sample; Shannon and Simpson indexes indicated the community diversity [50]. The indices were summarized per group of nestlings and adult crested ibises as the means and standard deviations. One-way ANOVA test was applied to evaluate significant differences of bacteria abundance and alpha diversity between different nestling groups and adult groups at different ages. Welch’s t-test was used to evaluate significant differences of bacteria abundance and alpha diversity between normal and sterile crested ibises. Cluster analyses were done by unweighted pair group method with arithmetic mean (UPGMA), non-metric multidimensional scaling (NMDS), and principal coordinate analysis (PCoA) [49]. The significance of beta diversity between the groups was evaluated by weighted and unweighted UniFrac distances. Analysis of Similarity (ANOSIM) was used to test whether the gut microbiota were significantly different between different groups. Correlation analysis was performed to reveal the association of various genera and the age of the nestlings by MATLAB. The microbial functional prediction was conducted by PICRUSt [51]. The Kyoto Encyclopedia of Genes and Genomes (KEGG) alignments were based on the predicted genes and their functions in phylogenetic analysis with Welch’s t-test (two-sided) and Benjamini–Hochberg FDR method [52]. Differentially abundant bacteria determined by linear discriminant analysis coupled with effect size (LEfSe) analysis using the Kruskal-Wallis test with linear discriminant analysis (LDA) score > 4 [53]. The Pearson’s r correlation and Spearman’s rank correlation were performed using the METAGENassist web server tool. The linear mixed effect models were used to evaluate the effects of sex, individual variability, age, and type of cage on gut microbiota in SPSS [54]. Venn diagrams were constructed using Venny [55]. Box plots and bar charts were generated using Graphpad Prism 8. Heatmap were constructed using the pheatmap package in R.

## Results

In total, 8,918,682 sequences were generated with an average length of 253 bp for each sequence **(S3 Table)**. Goods coverage index was > 99% in all samples, which suggests a good coverage of the libraries. The rarefaction curves tended to be flat, indicating that the sequencing data is reasonable and the specific sample has been sufficiently sequenced to represent its identity **(S1 Fig)**. More than 99% of OTUs were classified as bacteria, the rest were Archaea and eukaryotes. We excluded the Eukarya and Archaea from all analyses. For 72 samples of nestlings, 5,493,118 effective sequences were obtained with an average of 76,293 per sample and an average sequence length of 253 bp. In total, 7167 kinds of OTUs were produced, belonging to 59 phyla, 136 classes, 208 orders, 408 families, 1006 genera, and 560 species. An average of 74,937 taxonomic OTUs per sample were generated with a maximum of 87,349 and a minimum of 42,253.

For 12 adult samples, 886,238 effective sequences were generated. The average sequence number of each sample was 73,853 and the average sequence length was 253 bp. In total, 2566 kinds of OTUs were produced. These belonged to 38 phyla, 95 classes, 152 orders, 283 families, 627 genera, and 295 species. An average of 72,341 taxonomic OTUs per sample were generated with a maximum of 85,837 and a minimum of 54,301.

In 11 adults that were sterile (n = 3) or normal (n = 8), 880,003 effective sequences were obtained. The average sequence number of each sample was 73,334 and the average sequence length was 253 bp. In total, 3981 kinds of OTUs were produced, with a total of 776,396. It belongs to 43 phyla, 100 classes, 157 orders, 315 families, 697 genera and 344 species. An average of 73,743 taxonomic OTUs per sample were generated with a maximum of 85,837 and a minimum of 63,352.

### Gut microbial composition of nestlings

**Fig 1:**
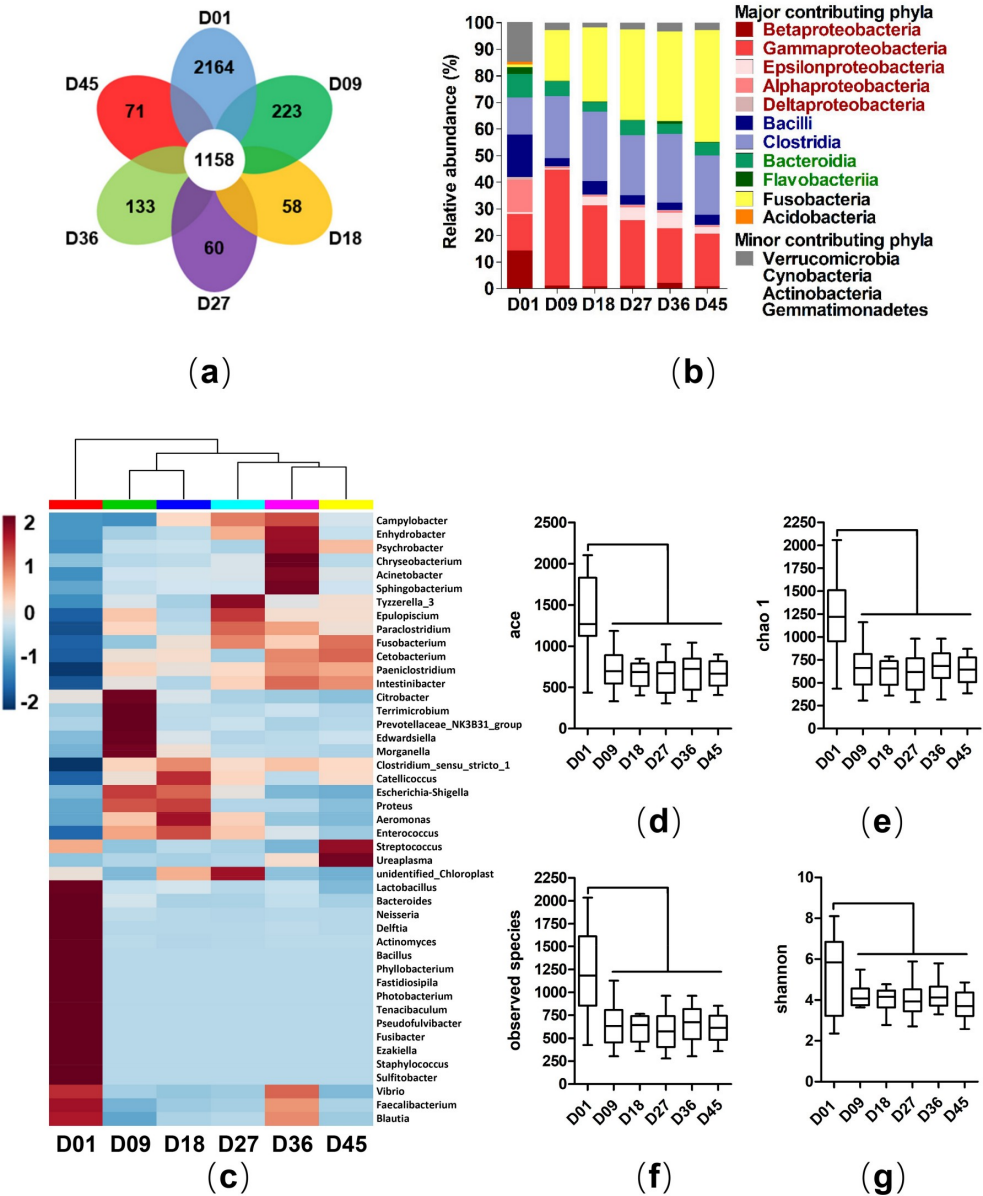
Structure and alpha diversity of gut microbiota of crested ibis nestlings at different ages. (a) Shared and unique OTUs at different ages. (b) Bar plot shows the relative abundance of bacterial phyla. Proteobacteria was replaced by five subclasses (alpha, beta, delta, epsilon, and gamma). Firmicutes was replaced by two subclasses (Bacilli, Clostridia). Bacteroidetes was replaced by two subclass levels (Bacteroidia, Flavobacteria). In the top 10 dominant phyla, major contributing phyla were defined as average relative abundance > 1%, and the rest were minor contributing phyla. (c) Relative abundance of core genera showed by heatmap. (d-g) Box plots show alpha diversity in different age groups of nestlings for four different dissimilarities: (d) ace, (e) chao 1, (f) observed species, (g) shannon.

To compare the composition of the identified community members in nestlings at different ages, we performed analysis at three phylogenetic levels: phylum, genus, and OTU. At the OTU level, 1158 microbial OTUs were common in all the age groups. Notably, there were 2164 unique OTUs in samples from D01, while there were only 58–223 unique OTUs in samples from all the other age groups **(Fig 1A)**.

At the phylum level, the four dominant phyla, Proteobacteria (34.70%), Firmicutes (28.40%), Fusobacteria (26.26%), and Bacteroidetes (6.50%) made up 96% of the total 59 identified phyla. Actinobacteria (0.93%), Tenericutes (0.37%), Verrucomicrobia (0.34%), Cyanobacteria (0.33%), Acidobacteria (0.29%), and Gemmatimonadetes (0.14%) made up around 3% of the total phyla identified **(Fig 1B) (S4 Table)**. ANOVA was applied to test the effects of age (D01 vs. D09 vs. D18 vs. D27 vs. D36 vs. D45) on the relative abundance of the identified phyla. The bacterial phyla of D01 were clearly distinguished from the other age groups, mainly in the four dominant phyla. We observed that with the increase of age, the abundance of Proteobacteria decreased gradually, which was 41.98% in D01, 45.90% in D09, 35.31% in D018, 31.51% in D27, 29.52% in D36, and 23.84% in D45 (P < 0.01). D01 mainly comprised of Betaproteobacteria (14.24%), Gammaproteobacteria (13.89%), and Alphaproteobacteria (12.18%). Samples from other age groups were mainly composed of Gammaproteobacteria (D09: 43.64%, D18: 30.33%, D27: 24.58%, D36: 20.53%, D45: 19.69%). The abundance of Fusobacteria significantly increased between D01 (1.16%) and D09 (19.05%) (P < 0.001). The abundance of Fusobacteria among D18, D27, D36, and D45 was 27.81%, 33.92%, 33.56%, and 42.05%, respectively. Additionally, the abundance of Firmicutes was comparable in all the age groups ranging from 26.27% to 31.28%. The dominant bacterial class of D01 differed significantly from the other age groups. Bacilli (15.97%) and Clostridia (13.83%) were the dominant classes in D01. Unlike D01, the samples from other age groups showed significantly higher abundance (P < 0.05) of Clostridia (D09: 23.23%, D18: 26.08%, D27: 22.50%, D36: 26.05%, and D45: 22.27%). We also observed significant dissimilarity in the abundance of Bacteroidetes between D01 and other age groups (D01: 12.02%, D09-D45: 5.15%-6.10%, P < 0.05) **(S4 Table)**.

At the genus level, we defined the core bacterial microbiota as the genera with an average relative abundance of more than 0.1%, resulting in 45 genera altogether. The heat map of core genera showed that D01 were clearly distinguished from those of other age groups (**Fig 1C**). Out of the 45 genera, 19 genera belonged to Proteobacteria, 17 genera belonged to Firmicutes, three genera belonged to Bacteroidetes, and six genera belonged to other distinct phyla. Among the 45 core genera, 12 genera were significantly enriched in D01 than other groups (P < 0.05), whereas 10 genera were significantly depleted in D01 as compared to the bacterial abundance in other groups (P < 0.05) **(S4 Table)**. Moreover, we observed that the abundance of three potential pathogens (*Escherichia-Shigella* [56, 57], *Edwardsiella* [58], and *Chryseobacterium* [59]) in D01 significantly differed from other groups: the abundance of *Edwardsiella* and *Chryseobacterium* were significantly higher in D01 than other groups while the abundance of *Escherichia-Shigella* were significantly lower in D01 than other groups **(S4 Table)**.

To identify specific taxa that are characteristic of different age groups, which could be used as potential biomarkers in the future, we carried out LEfSe analysis on the pooled taxa with LDA scores > 4. A total of 14 unique genera were found among the different groups; seven potential biomarkers were present in D01, three were present in D18, two were present in D45, and one was present in the remaining groups **(S2A Fig)**. A cladogram analyzed at the family level identified a total of 16 potential biomarkers in all the groups with a maximum number of biomarkers in D01 **(S2B Fig)**.

Pearson’s r correlation was performed to analyze the correlation of bacterial genera at various age-groups **(S3 Fig)**. The correlation coefficients ranged from 0.608 to 0.997 in all the comparisons. The correlation coefficient (0.997) was the highest between D27 and D36, while the correlation coefficient (0.608) between D01 and D45 was the lowest **(S5 Table)**.

### Alpha diversity and beta diversity of nestlings

**Fig 2:**
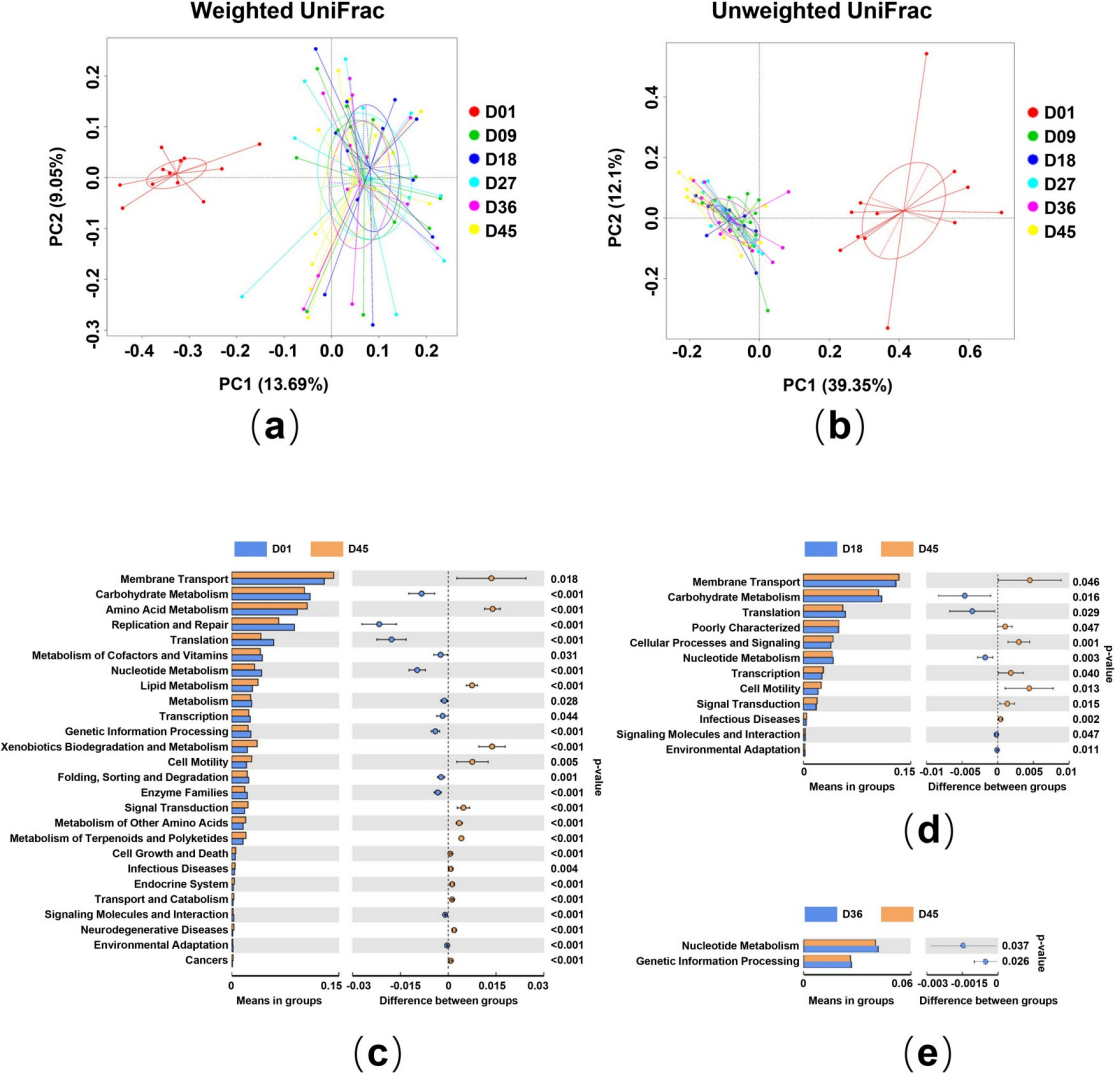
Exposition of bacterial beta diversity and microbial metabolic pathways at different ages of crested ibis nestlings. (a, b) Microbiota separation based on principal coordinates analysis (PCoA) from (a) unweighted UniFrac distances and (b) weighted UniFrac distances. The default ellipse was the standard deviation of the group medians. (c-e) Differences in microbial functions between different ages based on KEGG functional categories predicted by PICRUSt. (c) D01 vs. D45; (d) D18 vs. D45; (e) D36 vs. D45. The extended error bar plot denotes the difference in the mean proportion of microbial metabolic pathways between the groups along with the associated confidence interval of the effect size and the p-value of Welch’s t-test (P < 0.05).

For community evenness and diversity measure, we observed a significant difference between D01 and any other group (**Fig 1D–1G**). The ace, chao1, shannon, and observed species were significantly higher in D01 (shannon: P < 0.05; ace, chao1, observed species: P < 0.001), while the other groups showed extremely comparable diversity and evenness measures **(S6 Table)**. To evaluate beta diversity, PCoA was used to display general similarities in bacterial community structures among the different samples. PCoA analyses indicated that samples of D01 were clearly distinguished from those of other age groups, based on both weighted and unweighted UniFrac distances (**Fig 2A and 2B**). ANOSIM was performed to statistically support the clustering of the bacterial communities in the PCoA analyses (P < 0.001), and it also showed that the bacterial microbiota of D01 significantly differed from the other groups. Moreover, the bacterial microbiota of D09 significantly differed from D01, D27, D36, and D45 **(S7 Table)**.

### Microbial functional performance of nestlings

We investigated the functional capacity of the gut microbiota present in the different age groups of nestlings. We identified 41 abundant secondary metabolism pathways, wherein 34 pathways revealed a significant difference between D01 and D45 (**Fig 2C**), 12 pathways revealed a substantial difference between D18 and D45 (**Fig 2D**), and two pathways revealed a significant difference between D36 and D45 (**Fig 2E**). The results indicated that there were fewer differences in metabolism pathways between different age groups as the nestlings became older. In all the identified secondary metabolism pathways, membrane transport (13.32%), carbohydrate metabolism (10.54%), amino acid metabolism (9.34%), replication and repair (8.28%), and energy metabolism (5.55%) were the top five dominant secondary metabolism pathways. The microbiota in D01 had a high relative abundance of functional capacities involved in substance metabolism, cellular processes, and signaling, while genetic information processing, nucleotide metabolism, replication, and repair were enriched in the microbiota harbored in D45 (**Fig 2C**).

### Profiles of gut microbiota in adult crested ibis

**Fig 3:**
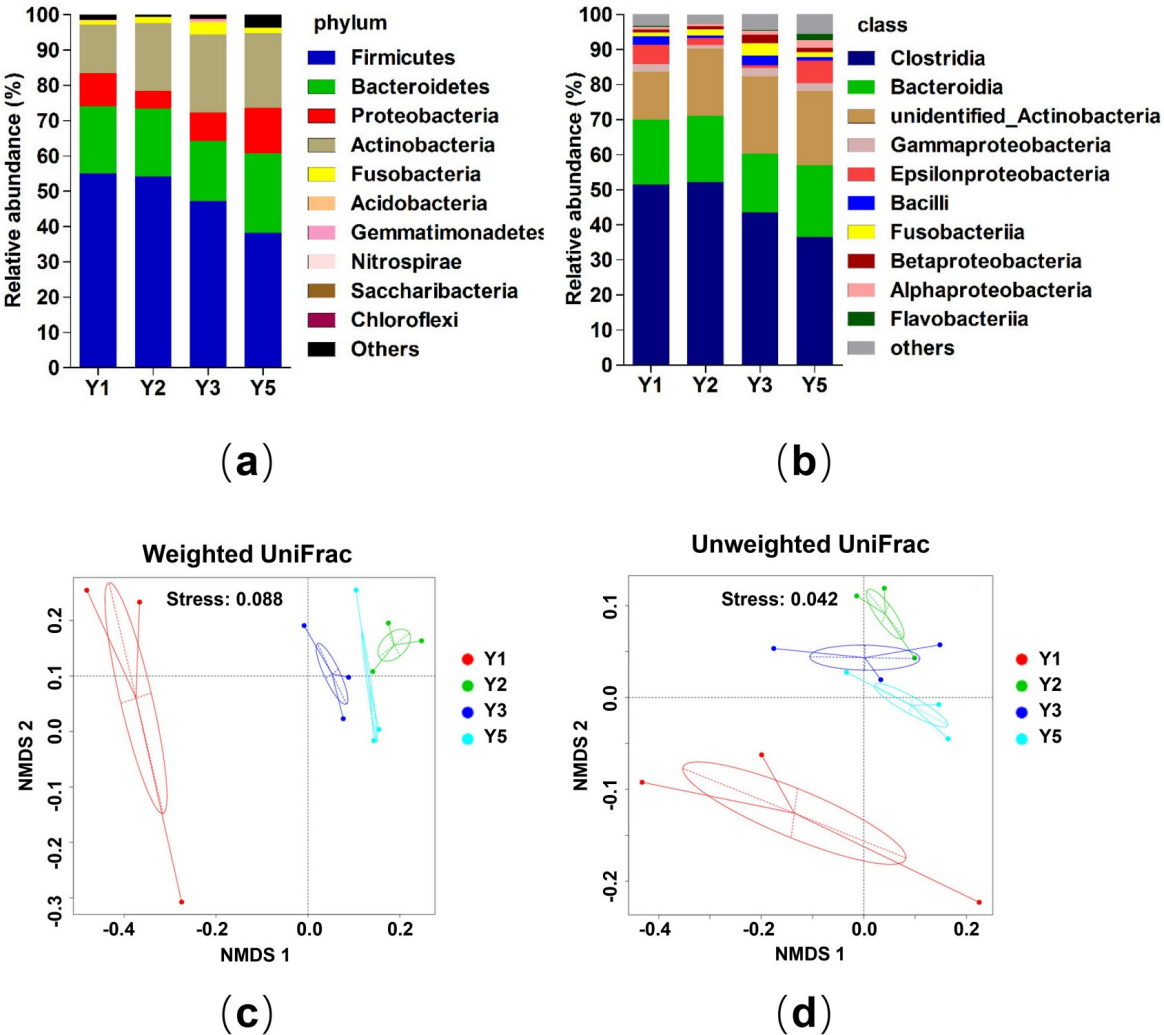
Composition and beta diversity of gut microbiota in adult crested ibis. (a, b) Relative abundance of top 10 bacterial taxa at (a) phylum level, (b) class level. (c, d) Microbial beta diversity of adults with a non-metric multidimensional scaling (NMDS) plot exhibiting distance among different ages. NMDS was generated using (c) unweighted UniFrac dissimilarity and (d) weighted Unifrac metrics. The default ellipse was the standard deviation of the group medians.

Among all the identified phyla in the gut microbiota of adult crested ibises, the predominant phyla accounting for more than 98% of the total microbiota were Firmicutes (48.69%), Bacteroidetes (19.36%), Actinobacteria (19.07%), Proteobacteria (8.86%), Fusobacteria (1.91%), and Acidobacteria (0.16%) (**Fig 3A**) **(S8 Table)**. There was no significant difference in the abundance of all phyla except Actinobacteria among various ages, which showed significantly lower abundance in the Y1 group than any other age groups. We found that Clostridia, Bacteroidia, and unidentified Actinobacteria were the three top dominant bacterial classes (**Fig 3B**) **(S8 Table)**. We also compared the difference in core genera at different ages (**S4A Fig**) and identified a total of 51 core genera, which mainly belonged to Firmicutes, Actinobacteria, and Proteobacteria. The genera, such as *Varibaculum* and *Peptococcus* had higher abundance in the Y2 group as compared to Y3 (P < 0.05). Moreover, *Fastidiosipila*, *Ezakiella*, *Actinomyces*, and *Varibaculumwere* were significantly depleted in the Y1 than Y2 group **(P < 0.05)**. Genera level correlations among microbes in each age-group indicated that most genera were negatively correlated **(S4B Fig)**. The alpha diversity indices displayed no significant difference among the four groups **(S9 Table)**. The NMDS analyses showed that samples of one-year-old crested ibises were more diverse than all other samples together. (**Fig 3C and 3D**).

### Comparison of gut microbiota between normal and sterile crested ibis

**Fig 4:**
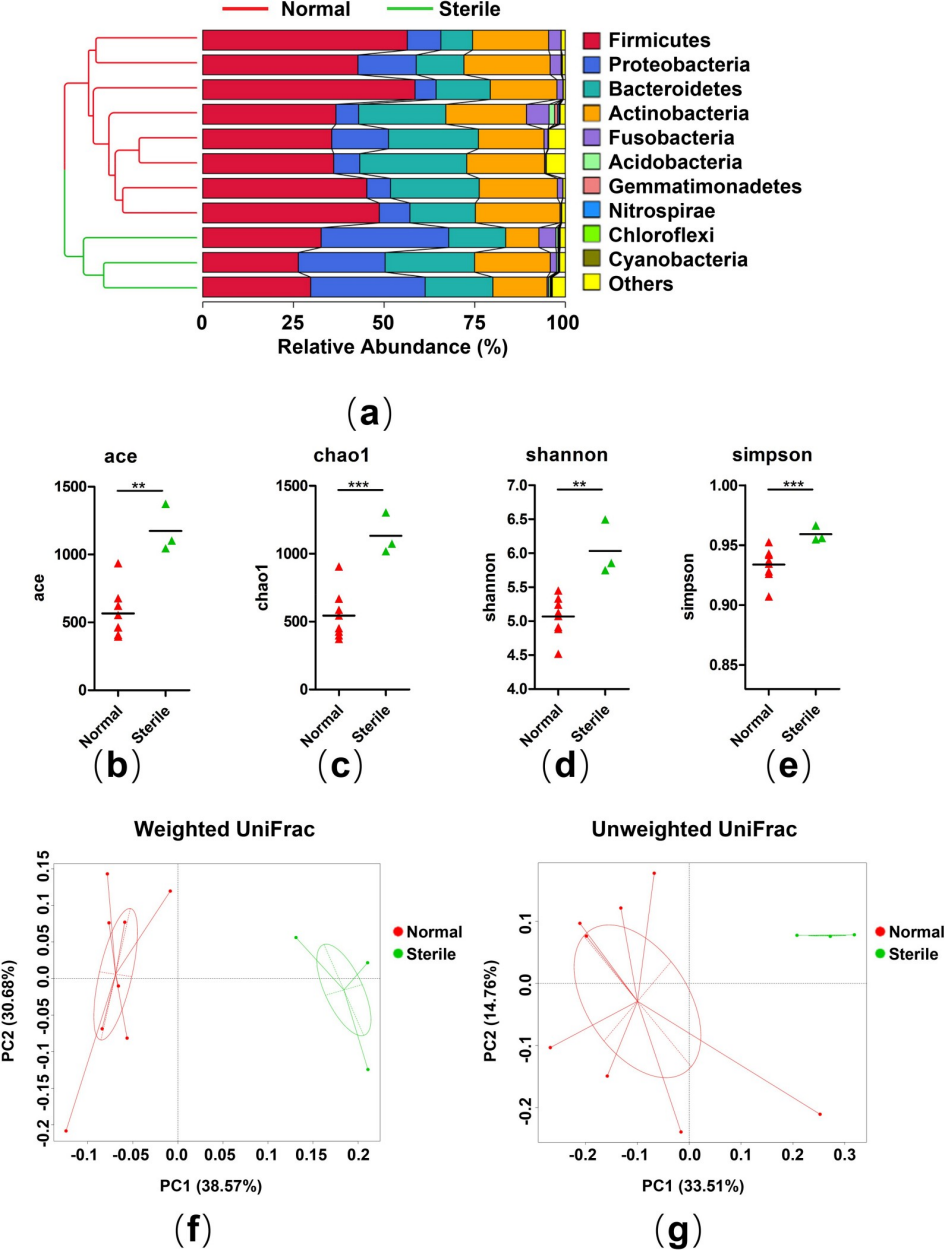
Comparison of gut microbiota between sterile and normal crested ibises. (a) The tree (left) shows the hierarchical clustering of the samples based on Bray-Curtis dissimilarity. The bar chart (right) shows the relative abundance of bacterial phyla in each individuum. The trees have different colors indicating the groups. (b-e) The community richness indicated by Ace (b), Chao1 (c), Shannon (d), and Simpson indices (e). Significance level: ** P < 0.05; *** P < 0.01. The significance of alpha diversity between groups was evaluated by the Welch’s t-test. (f, g) The beta diversity of bacterial communities was associated with different groups. Principal coordinate analysis (PCoA) was generated using unweighted (f) and weighted (g) Unifrac metrics. The default ellipse was the standard deviation of the group medians.

To understand the correlation between gut microbiota and reproductive capacity of adult crested ibises, we compared the microbiota of normal and sterile crested ibises. The hierarchical clustering algorithm, UPGMA, was used to examine the identified OTUs from all the samples based on Bray-Curtis dissimilarity. We observed the strong clustering of bacterial communities in terms of reproductive capacity (**Fig 4A**). Moreover, alpha diversity indices, including Ace, Chao1, Shannon, and Simpson indices were also measured (**Fig 4B–4E**). Both community diversity indices (shannon, simpson) and community richness indices (ace, chao1) of microbes in sterile crested ibises were significantly higher than those in normal crested ibises **(S10 Table)**. PCoA analyses revealed that samples were clustered into two groups based on reproductive capacity (**Fig 4F and 4G**). Moreover, ANOSIM indicated the bacterial community structures of sterile crested ibises were clearly distinguished from those of healthy crested ibises (unweight: R = 0.7621, P = 0.018; weight: R = 0.6278, P = 0.005). We predicted a functional map to compare the differences of bacterial function between the two groups by using PICRUSt. Based on level 2 of KEGG pathway analysis, the abundance of 21 pathways differed significantly between normal and sterile crested ibises (**S5A Fig**). In the normal crested ibises, replication and repair, energy metabolism, translation, nucleotide metabolism, and metabolism of cofactors and vitamins were significantly (P < 0.05) enriched. However, we detected a high relative abundance of membrane transport, lipid metabolism, cell motility, and xenobiotics biodegradation and metabolism in sterile crested ibises.

### Proteobacteria: A potential biomarker of reproductive capacity

We assessed differences in the bacterial community at four levels in sterile and normal crested ibises, i.e., phylum, class, family, and genus levels, to identify gut bacterial potential biomarkers related to reproductive capacity. At the phylum level (**Fig 4A**), Firmicutes was significantly (P < 0.05) depleted in the sterile crested ibises (29.60%), whereas Proteobacteria were significantly (P < 0.05) enriched in this group (45.00%) as compared to the normal crested ibises, which were composed of 45.00% Firmicutes and 9.41% Proteobacteria. The relative abundance of Bacteroidetes (normal: 19.71%, sterile: 19.68%), Actinobacteria (normal: 21.23%; sterile: 14.99%), and Fusobacteria (normal: 2.20%, sterile: 2.25%) were comparable in both the groups **(S11 Table)**.

At the class level **(S5B Fig)**, Alphaproteobacteria (P < 0.05), Betaproteobacteria (P < 0.05), and Gammaproteobacteria (P < 0.05) had higher relative abundance in the sterile crested ibises as compared to that in the normal crested ibises. At family level (**S5C Fig**), Family_XI (29.77%), *Porphyromonaceae* (16.63%), *Ruminococcaceae* (11.18%), and *Actinomycetaceae* (15.50%) were the top four dominant families in the normal group, accounting for 73.08% of the total families; however, Family_XI (11.14%), *Poromonaceae* (14.28%), *Actinomycetaceae* (11.25%), and *Enterobacteriaceae* (9. 59%), were the top four dominant families in the sterile group, accounting for 46.26% of the total families. The relative abundance of Family XI was significantly lower (P < 0.05) in the sterile group than in the normal group, while the relative abundance of *Enterobacteriaceae*, *Peptostreptococcaceae* and *Comamonadaceae* was significantly higher **(S11 Table)**.

**Fig 5:**
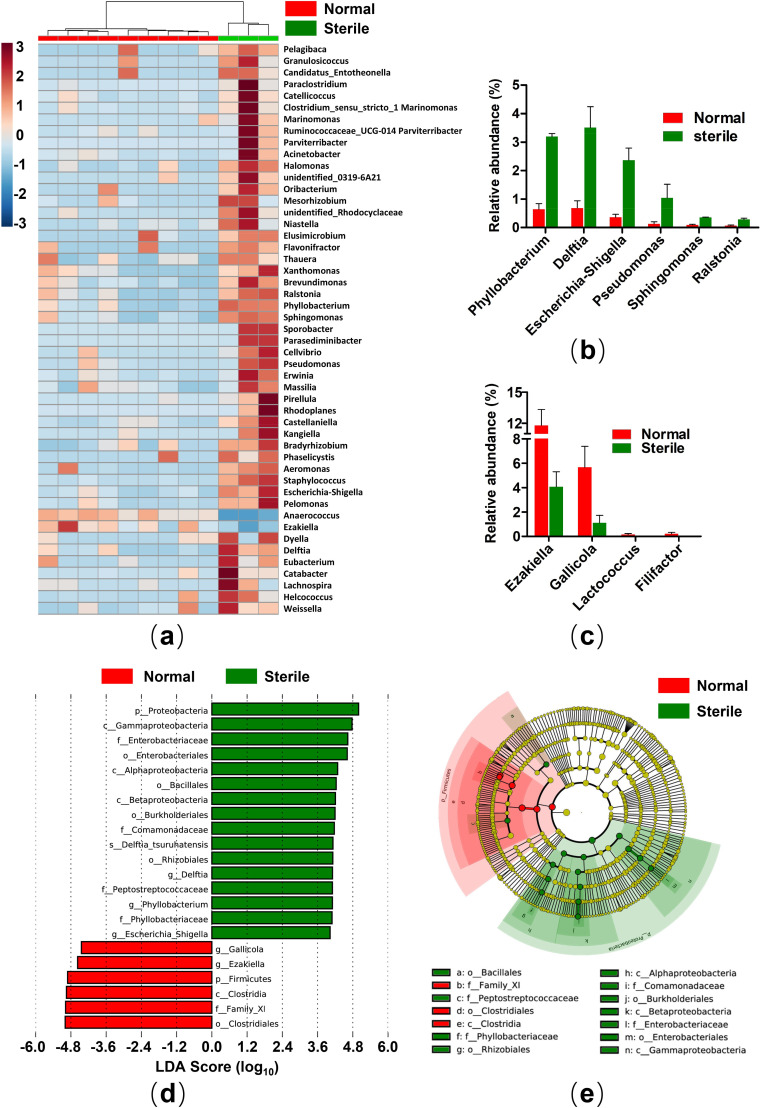
Potential biomarkers of sterile crested ibises. (a) Heatmap shows the relative proportion of each core bacterial genus. (b, c) The relative abundance of the core bacteria at the genus level with a significant difference. The bacteria belonged to (b) Proteobacteria and (c) Firmicutes at the phylum level. (d) Differentially abundant bacteria determined by LEfSe analysis using the Kruskal-Wallis test (P < 0.05) with LDA score > 4. (e) Cladogram shows the differentially abundant bacteria at different taxonomic levels. The size of each node indicates their relative abundance. The letters at the beginning of taxa names indicate: p, phylum; c, class; o, order; f, family; g, genus.

At the genus level (**Fig 5A**) **(S11 Table)**, 49 core genera were identified. The heat map of core genera in all the samples showed a complete clustering according to reproductive capacity. We observed that 24 core genera belonged to Firmicutes, and 13 core genera belonged to Proteobacteria, and the remaining 12 genera belonged to other phyla. For Proteobacteria (**Fig 5B**), 6 out of the 13 genera were significantly enriched in the sterile crested ibises as compared to the normal crested ibises. *Escherichia-Shigella* [56, 57] and *Pseudomonas* [60] were the potential pathogens. In contrast, for Firmicutes (**Fig 5C**), 4 out of the 24 genera exhibited lower abundance in the sterile crested ibises than in the normal crested ibises. Moreover, the LEfSe analysis of the taxa characterized differences between the two groups (**Fig 5D and 5E**). The result is consistent with the above conclusion. Proteobacteria contributed greatly to the differentiation of microbiota between normal and sterile crested ibises. The representative bacterial orders of Proteobacteria were Enterobacteriales and Pseudomonadales.

#### The other effects on gut microbiota of nestlings and adults crested ibises

To study the effects of sex, individual variability, age, and type of cage on gut microbiota of crested ibises, microbiota characteristics (taxonomic composition and alpha diversity indexes) were tested with linear mixed effect models including different fixed factors: (1) for nestlings, sex, individual variability, and type of cage were analysed (**S12 Table**); (2) for adults, sex was analysed (**S13 Table**); (3) for normal and sterile adults, sex and age were analysed (**S14 Table**). There were no significant differences on them.

## Discussion

### Establishment of gut microbiota in nestlings

Intestinal microbes co-evolve in highly dynamic communities, and their composition changes with the age of the host [61]. For instance, in humans, the composition of gut microbiota changes dramatically from birth to adulthood, until it reaches a stable community structure [62, 63]. Two critical factors influence the establishment of mature microbial flora: the transformation of diet and changes of oxygen content in the enteric cavity of the host. Besides, with the maturation of innate or adaptive immune mechanisms in the intestinal mucosa, the microbial community composition also varies, and the immune mechanisms and bacterial community correlate with each other [10].

In this study, we observed that the community structure of gut bacterial flora of D01 nestlings differed significantly from that of the nestlings of other age groups. Richness and diversity of D01 nestlings were higher than those of other age groups; the number of unique OTUs in D01 nestlings was 9–37 times higher than that of nestlings of other age groups. These results are in contrast to findings from most previous studies, which have reported that gut microbial diversity increases continuously from birth to adulthood in humans, chickens, and pigs [64–67]. However, previous studies have also described gut microbial diversity-trend similar to our results in chicken and arctic shorebirds [12, 68]. The host diet and nutritional habit can also affect the host’s microbial community composition. In humans, the transition from breastfeeding to mixed feeding, and ultimately to an adult diet, leads to significant changes in the community structure of intestinal microorganisms [62, 69]. Previous studies have shown that the digestive system of chicks during the first week is reduced, which causes less absorption of nutrients from the feed. About 40% of nutrients in eggs are transferred to the yolk sac of newborn chicks, which is the primary source of nutrients for newly hatched chicks. The nutrition of 1-3-days-old chicks is acquired entirely from the yolk sac; 5-7-days-old chicks undergo a transition from yolk sac nutrition (endogenous) to feed nutrition (exogenous); the nutrition of 8-14-days-old chicks is acquired entirely from feed [70, 71]. Therefore, we speculated that since the yolk sac is the main source of nutrition for D01 nestlings, which is exhausted after 2–3 days, only the bacteria that can use nutrients from feed or bacterial metabolites could survive after the yolk sac is exhausted; thus, the bacterial diversity decreased. Besides, the functions of microbes present in D01 nestling were mainly related to material metabolism and cell processes, while the purpose of intestinal bacterial flora in D45 nestlings was chiefly related to genetic information processing. Mackie (1999) showed that the concentration of protease, amylase, and lipase increases (in different ratios) with the increase in the age of chickens [72]; thus, it is possible that the primary function of bacterial flora in D01 was substance metabolism due to lower concentration of various digestive enzymes.

In this research, the succession of the dominant community reflected a dynamic process, wherein in the gut microbiota of crested ibises changed from aerobic to facultative anaerobic flora. Although the total abundance of Firmicutes was comparable in the fecal microbiota of nestlings at different ages, Firmicutes in D01 group was mainly composed of 50% anaerobic Clostridia and 50% facultative or obligate aerobic Bacilli, while Firmicutes in other age groups mainly consisted of anaerobic Clostridia (90% of the total bacteria). The abundance of the class Clostridia, most of which were strict anaerobes, increased, while that of Proteobacteria, which were aerobes and facultative anaerobes, decreased gradually as the age of the nestlings increased. The intestinal environment is known to maintain an ecological pattern with more anaerobic bacteria and less aerobic bacteria [72]. However, at birth, the intestine is filled with a substantial amount of oxygen, which is conducive to the first colonization of aerobic bacteria or facultative anaerobic bacteria. The growth and reproduction of these bacteria consume most of the oxygen, reduce the intestinal redox potential, and create an environment for colonization by anaerobic bacteria [73, 74]. Furthermore, anaerobic bacterial metabolites, various volatile fatty acids, and lactic acid inhibit the growth of aerobic bacteria. As a result, the dominant aerobic bacterial community is transformed into a facultative anaerobic bacterial community. Previous studies have also shown that Firmicutes increase, while Proteobacteria decrease, inversely proportional to the age in chickens and wild house sparrow nestlings [75, 76].

Furthermore, the intestinal immune system has a regulatory effect on the intestinal flora. In our study, the change of Proteobacteria and Clostridia could also be related to the immune system, which indicated the gradual maturity of the gut microbiota in crested ibises. Experiments in humans [74], chickens [22], and mice [77] have demonstrated that the abundance of the Proteobacteria is higher in newborns than in adults. Researchers reported that the gradual maturity of the gut microbiota is characterized by a significant decrease in the proportion of Proteobacteria [77]. Thus, it can be speculated that in the early stages of bacterial colonization, the immune system is incomplete, and the abundance of Proteobacteria is high; however, as the immune system matures, the abundance of Proteobacteria decreases. We also observed that the abundance of Clostridia in D01 nestlings was the lowest. Researchers have found that Clostridia plays a protective role in the intestinal tract of human newborns. Clostridiales are absent in the mice neonatal microbiota and they increase with increase in the age of mice. Due to *Clostridium* colonization, several pathogenic bacteria that can cause intestinal infections are inhibited to varying extents [78].

We also observed that Fusobacteria were significantly enriched in crested ibis nestlings. These results are in accordance with previous studies, which have identified numerous Fusobacteria in the intestines of carnivorous birds. In vulture intestines, one-third of the intestinal gut microbes belongs to Fusobacteria [79, 80]; in penguin intestines, more than half of the gut microbiota belongs to Fusobacteria [81, 82]. Previously, members of Fusobacteria were mainly associated with pathogenic bacteria, but the study on vultures indicated that members of Fusobacteria might also be beneficial. More studies are required to explore the function of Fusobacteria in birds.

This study displayed that composition, alpha diversity, and function of gut microbiota of crested ibis nestlings stabilized with an increase in age. In this process, both active recruitment and passive environmental selection were involved. After the initial colonization, only the species most suitable for intestinal conditions can be preserved and propagated, and the host actively recruits bacteria through the immune system. Thus, the colonization of flora at birth and the succession of flora after birth could be influenced by many factors, such as birth mode, feeding mode, antibiotic application, hygienic conditions, and geographical environment [3, 8, 18]. However, no consistent results about the effects of these factors have been obtained. In this study, we have found that type of cage and sex have no effects on the gut microbiota between nestlings under 45 days old. A more comprehensive study on flora colonization and succession is required.

### Gut microbiota of adult birds

The identification of gut microbiota is the first step in showing that stable community structure of gut microbiota is vital for the health of the host [26, 27, 83]. This study focuses on captive crested ibises. We observed no significant difference in alpha diversity among crested ibises aged 1, 2, 3, and 5 years. The taxonomic profile of gut microbiota of adult crested ibis is more similar to that of chickens than that of humans, mammals, and other wild birds [18, 20]. The dominant bacterial phyla in the gut microbiota of crested ibis are Firmicutes, Bacteroidetes, and Actinobacteria, followed by Proteobacteria with low abundance. The abundance of Firmicutes in adult crested ibis is similar to that in mammals, humans, chickens, and wild birds accounting for approximately 50% of the total bacteria [18]. Firmicutes, the major energy sources for the host, can produce short-chain fatty acids, which are associated with the metabolism of sugars, fatty acids, polysaccharides, and complex carbohydrates [84, 85]. The crested ibis adults carry a lower proportion of Proteobacteria (9%) than chickens and other wild birds (25%). Potential pathogens in the adults belonged to Proteobacteria, such as *Salmonella*, *Escherichia*, *Helicobacter*, and *Campylobacter*, which have also been previously isolated from avian gut [86–89]. However, the function of Proteobacteria in birds remains undetermined and further studies are needed.

Due to the different sample types and sampling time in nestling and adults, we haven’t tested the differences among nestlings and adults in this study. While, we observed a significant difference in the gut microbial composition of crested ibis nestlings and adults. The dominant phyla in nestlings were Firmicutes, Proteobacteria, and Fusobacteria, whereas Firmicutes, Bacteroidetes, and Actinobacteria were the main phyla enriched in adult birds. The abundance of Firmicutes, Bacteroidetes, and Actinobacteria increased in adult crested ibises, while the abundance of Proteobacteria and Fusobacteria depleted. This result could be due to the change in environment and diet. The difference in the composition of microbiota observed in our study could be because after 45 days of age, the nestlings were put into new cages and fed on adult forage instead of juvenal fodder. However, further studies are required to establish the effect of environment and food changes on the intestinal microflora of crested ibises after 45 days of age.

### The association between gut microbiota and reproductive output

The mammalian gut microbiota is often assumed to be stabilized when the host reached maturity [90]. It is also known that microbial composition exerts an important influence on host health. This study indicated that sterile crested ibises had an increased abundance of Proteobacteria as compared to that in normal birds. Previous studies have suggested that an abnormal expansion of Proteobacteria in gut microbiota is a sign of intestinal dysbacteriosis and can be used as a potential diagnostic biomarker of intestinal diseases [77, 91, 92]. Furthermore, the abundance of *Enterobacteriaceae* in sterile crested ibises was significantly higher than that in normal crested ibises. Previously, it has been reported that the adaptor protein MyD88 is crucial in regulating intestinal flora of humans [93]. MyD88 can restrict the growth of *Enterobacteriaceae* but facilitates the growth of *Bacteroidaceae*. B cells have also been observed to limit the expansion of the *Enterobacteriaceae* [94]. Therefore, an increased abundance of *Enterobacteriaceae* in crested ibises could also indicate abnormalities in the immune system.

We observed the following correlations between the abnormal expansion of Proteobacteria and the reproductive system of the crested ibises. First, gut microbiota associated with hormone metabolites could affect reproductive capacity. Several studies have shown that changes in the intestinal microflora can cause metabolic syndromes, which are associated with diabetes, obesity, glucose intolerance, and infertility [32]. Reportedly, abnormal human gut microbiota can lead to an increase in serum insulin, which can directly affect ovaries, increase male hormone production, and interfere with normal follicular development [95]. Antwis et al. (2019) indicated that the variation in gut microbiota is a potential biomarker of reproductive health for endangered eastern black rhino because four genera of the gut microbiome are associated with hormone production and reproductive output [39]. Second, the pathogenic bacteria of the gut in crested ibis could have infected the reproductive system. In birds, the ureter and genital duct converge in the cloaca, and feces, urine, and germ cells are discharged through it. This physiological structure provides an opportunity for the intestinal microorganisms to enter the reproductive system, and can cause reproductive system diseases. In sterile crested ibises, opportunistic pathogenic bacteria, *Escherichia-Shigella* and *Pseudomonas*, were highly abundant than in the normal birds. The two genera can be potential biomarkers of reproductive health for crested ibises. White et al. (2010) reported that a wild population of kittiwakes was infected by sexually transmitted bacteria, which affected the bacterial diversity and composition of female birds [96]. Thus, in crested ibises, pathogens could also be transmitted during copulation.

Thus, this study reflects the origin of intestinal microbiota in crested ibises as well as the fragile structure of microbial flora. We also have identified an association between the gut microbiota and reproductive capacity of crested ibises, indicating that the composition or diversity of gut microbiota can be used as a biomarker for reproductive health in endangered wild animals. Moreover, animal feces can be non-invasively sampled; non-invasive detection of reproduction capacity provided an essential step for the expansion of the population of crested ibis. This study will also encourage studies on the gut microbiota of other endangered species in a non-invasive manner. However, further studies on gut microbiota are required for unraveling the mechanisms that affect reproductive success, to identify specific bacteria, and study hormone metabolites and their interactions.

## Supporting information

S1 Fig Rarefaction curve for each group. The x-axis indicates the number of sequencing bars randomly selected from a group, and the y-axis indicates the number of OTUs constructed based on the number of sequencing bars. (a) nestling crested ibises; (b) adult crested ibises; (c) healthy and sterile crested ibises.
(TIF)

S2 Fig Biomarkers of different ages in nestlings. (a) LEfSe analysis indicates differentially abundant bacteria as biomarkers determined using Kruskal-Wallis test (P <0.05) with LDA score > 4. (b) Cladogram shows the taxonomic hierarchical structure of the phylotype biomarkers identified between different ages. The circle on the left represents the distribution of different biomarkers between groups at different taxonomic levels. The list on the right shows the species with significant differences on the left. The letters at the beginning of taxa names indicate: p, phylum; c, class; o, order; f, family; g, genus.
(TIF)

S3 Fig Microbial correlation of different age-groups at phylum level. Pearson’s r correlation was performed to analyze the correlation of bacterial genera at various age-groups.
(TIF)

S4 Fig Composition and correlation of gut microbiota in adult crested ibises. (a) Heatmap shows relative proportion of core bacterial genera. (b) Correlation among the core bacterial genera.
(TIF)

S5 Fig Comparison of microbiota between sterile and normal crested ibises. (a) Significant difference in bacterial function between sterile and healthy crested ibises based on KEGG functional categories predicted by PICRUSt. The extended error bar plot denotes the difference in the mean proportion of microbial metabolic pathways between the groups along with the associated confidence interval of the effect size and the p-value of Welch’s t-test (P < 0.05). (b) Relative abundance of microbiota between the two groups at class level. (c) Relative abundance of gut microbiota between different groups at the family level.
(TIF)

S1 Table The feed formula of nestlings and adult crested ibises. In the growth stage of the nestlings during the period 1 day to 45 days old, a total of 6 sets of feed formula are used to meet the nutritional needs of different growth stages.
(XLSX)

S2 Table Background information on the crested ibis samples. The background information includes individual number, group name, sex, accession number, type of cage, and locality.
(XLSX)

S3 Table Sequencing information on the crested ibis samples. Preprocessing statistics and quality control of raw data: Raw PE, original data derived from the indicated primer pair; Combined, original data were assembled by overlapped sequence; Qualified, low‐grade quality and short‐length reads were filtered out to generate qualified data; Nochime, chimeric sequences were filtered out to generate effective sequences; Base, the base number of total data; AvgLen, average length of qualified tags; Q20, the percentage of bases with sequencing error rate < 1%; Q30, the percentage of bases with sequencing error rate < 0.1%; GC%, GC content; Effective%, the percentage of effective tags.
(XLSX)

S4 Table Significant differences between nestling groups at the phylum, class and genus levels. The abundance of microbiota was summarized per group of nestling crested ibises as the means, standard errors, and standard deviations. One-way ANOVA test was applied to evaluate significant differences.
(XLSX)

S5 Table The correlation coefficients of bacterial genera at various age-groups. (XLSX)

S6 Table Significant differences of alpha diversity between nestling groups. The alpha diversity indexes were summarized per group of nestling crested ibises as the means, standard errors, and standard deviations. One-way ANOVA test was applied to evaluate significant differences.
(XLSX)

S7 Table Differences in the bacterial community composition of nestling feces based on the similarity test of ANOSIM. (XLSX)

S8 Table Significant differences between adult groups at the phylum, class and genus levels. The abundance of microbiota was summarized per group of adult crested ibises as the means, standard errors, and standard deviations. One-way ANOVA test was applied to evaluate significant differences.
(XLSX)

S9 Table Significant differences of alpha diversity between adult groups. The alpha diversity indexes were summarized per group of adult crested ibises as the means, standard errors, and standard deviations. One-way ANOVA test was applied to evaluate significant differences.
(XLSX)

S10 Table Significant differences of alpha diversity between normal and sterile crested ibises. The alpha diversity indexes were summarized per group of normal and sterile crested ibises as the means, standard errors, and standard deviations. The Welch’s t-test was applied to evaluate significant differences.
(XLSX)

S11 Table Significant differences between normal and sterile crested ibises at the phylum, class, family, and genus levels. The abundance of microbiota was summarized per group of normal and sterile crested ibises as the means, standard errors, and standard deviations. The Welch’s t-test was applied to evaluate significant differences.
(XLSX)

S12 Table The effects of sex, individual variability, type of cage on gut microbiota of nestlings. The microbiota characteristics (taxonomic composition and alpha diversity indexes) were tested with linear mixed effect models to evaluate the effects of sex, individual variability, and type of cage on gut microbiota in nestlings.
(XLSX)

S13 Table The effect of sex on gut microbiota of adults. The microbiota characteristics (taxonomic composition and alpha diversity indexes) were tested with linear mixed effect models to evaluate the effect of sex on gut microbiota in adults.
(XLSX)

S14 Table The effects of sex and age on gut microbiota of normal and sterile crested ibises. The microbiota characteristics (taxonomic composition and alpha diversity indexes) were tested with linear mixed effect models to evaluate the effects of sex and age on gut microbiota in normal and sterile crested ibises.
(XLSX)
