# Supplementary figures and images for: Gut microbiota of endangered crested ibis: Establishment, diversity, and association with reproductive output
Source: PLoS One. 2021 Apr 23;16(4):e0250075. doi: 10.1371/journal.pone.0250075 (PMC8064547; doi:10.1371/journal.pone.0250075)

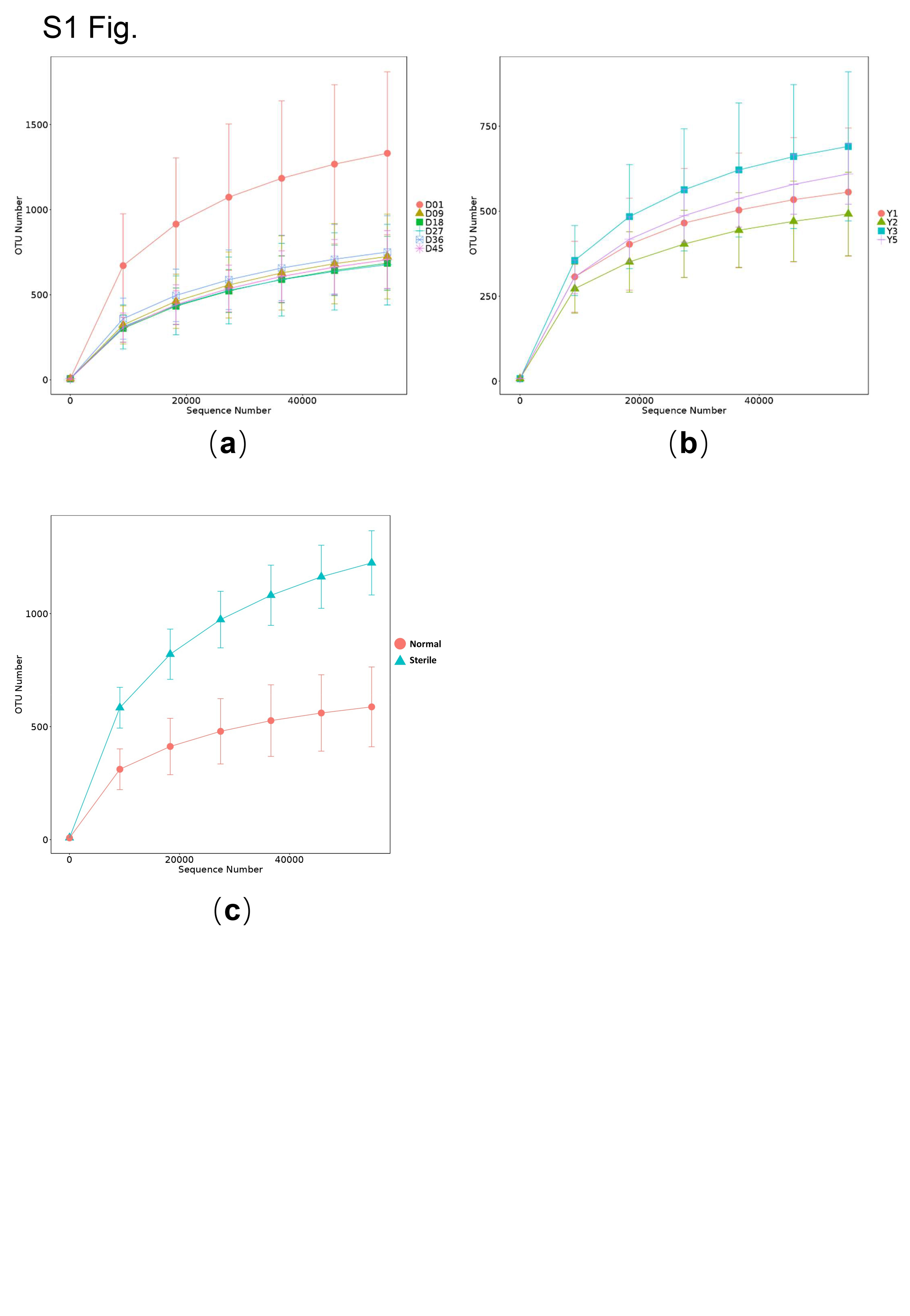

Supplement: S1 Fig — The x-axis indicates the number of sequencing bars randomly selected from a group, and the y-axis indicates the number of OTUs constructed based on the number of sequencing bars. (a) nestling crested ibises; (b) adult crested ibises; (c) healthy and sterile crested ibises. (TIF) [file pone.0250075.s001.tif]

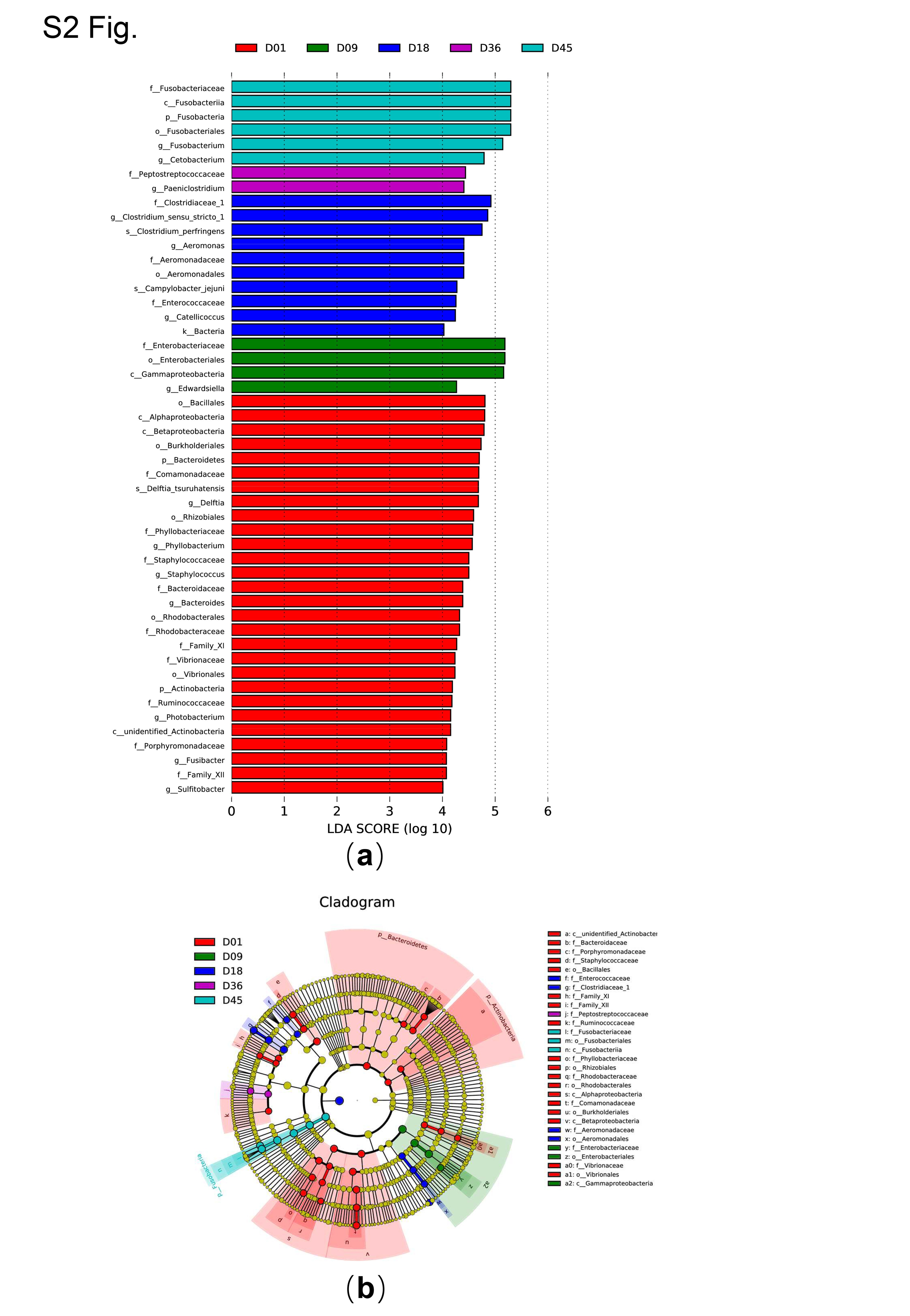

Supplement: S2 Fig — (a) LEfSe analysis indicates differentially abundant bacteria as biomarkers determined using Kruskal-Wallis test (P <0.05) with LDA score > 4. (b) Cladogram shows the taxonomic hierarchical structure of the phylotype biomarkers identified between different ages. The circle on the left represents the distribution of different biomarkers between groups at different taxonomic levels. The list on the right shows the species with significant differences on the left. The letters at the beginning of taxa names indicate: p, phylum; c, class; o, order; f, family; g, genus. (TIF) [file pone.0250075.s002.tif]

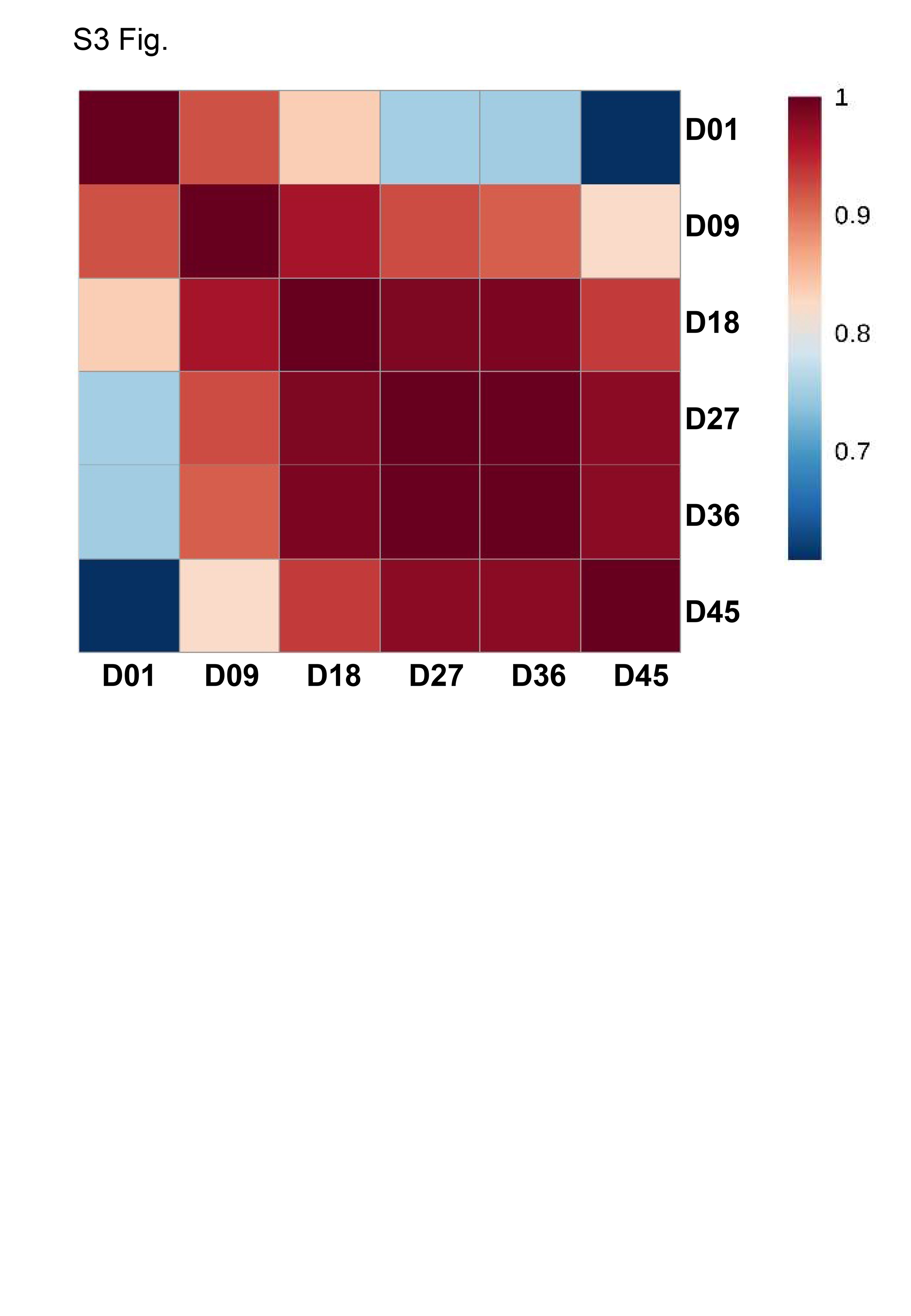

Supplement: S3 Fig — Pearson’s r correlation was performed to analyze the correlation of bacterial genera at various age-groups. (TIF) [file pone.0250075.s003.tif]

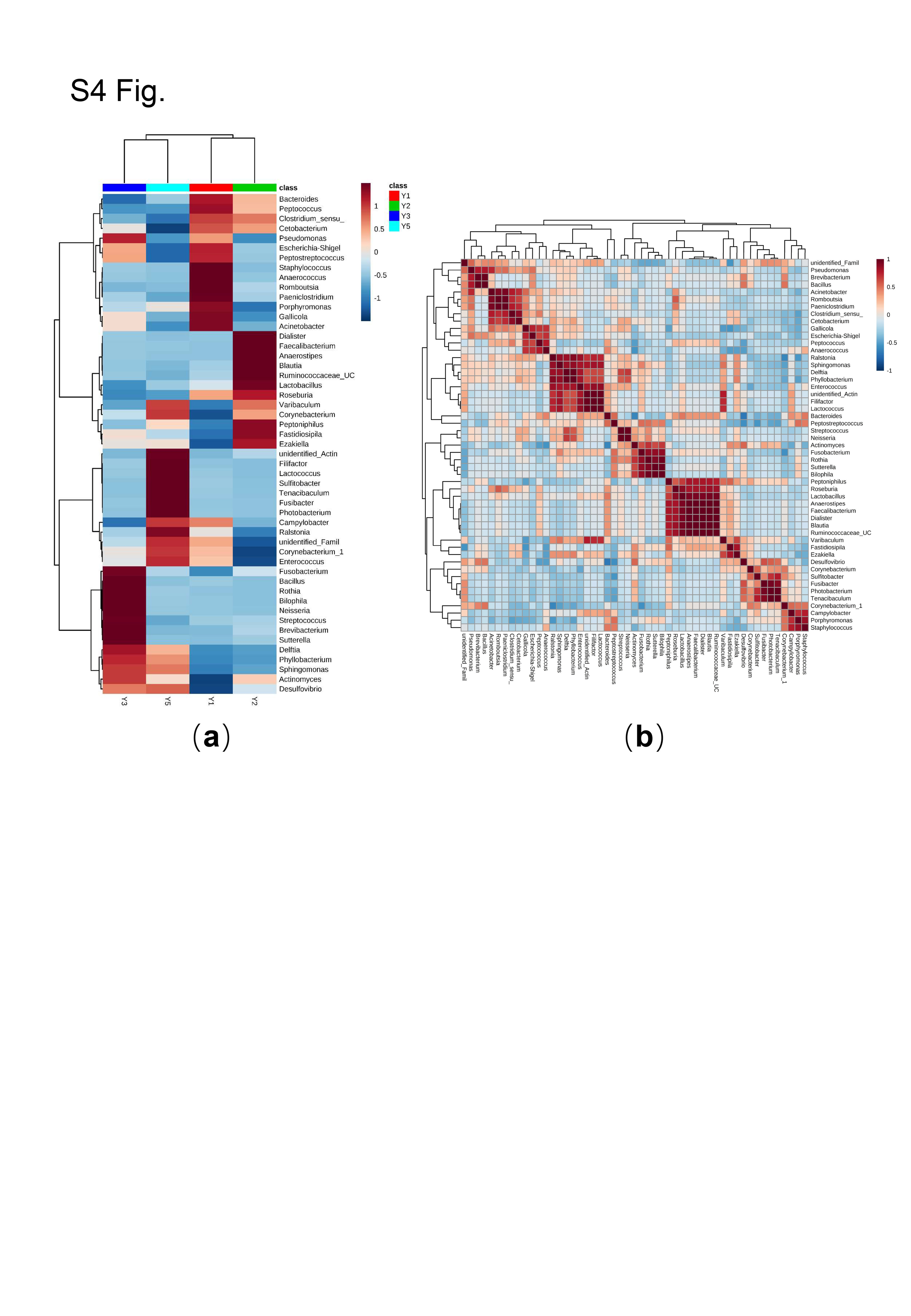

Supplement: S4 Fig — (a) Heatmap shows relative proportion of core bacterial genera. (b) Correlation among the core bacterial genera. (TIF) [file pone.0250075.s004.tif]

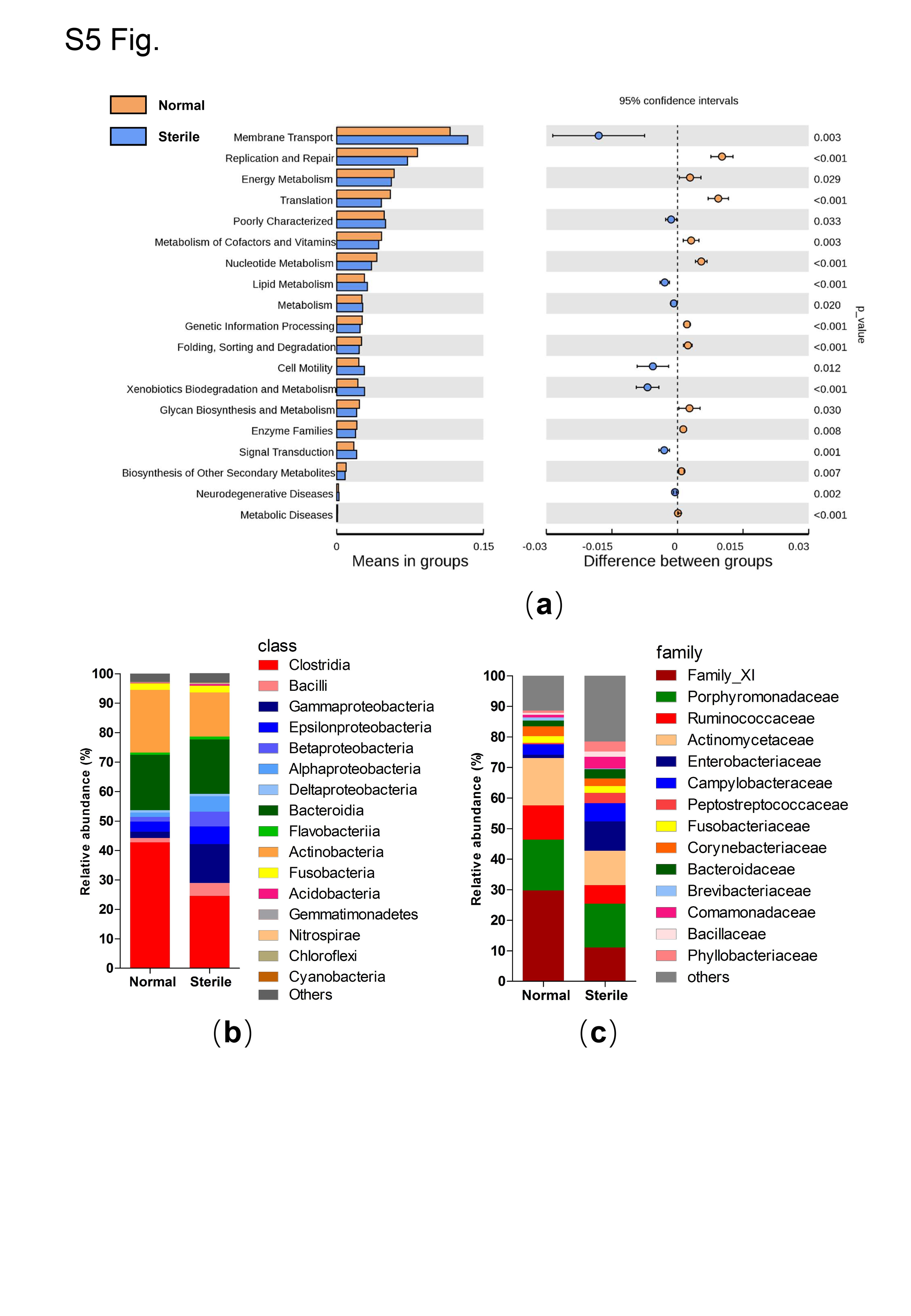

Supplement: S5 Fig — (a) Significant difference in bacterial function between sterile and healthy crested ibises based on KEGG functional categories predicted by PICRUSt. The extended error bar plot denotes the difference in the mean proportion of microbial metabolic pathways between the groups along with the associated confidence interval of the effect size and the p-value of Welch’s t-test (P < 0.05). (b) Relative abundance of microbiota between the two groups at class level. (c) Relative abundance of gut microbiota between different groups at the family level. (TIF) [file pone.0250075.s005.tif]
